# Characterization of the Antibody and Interferon-Gamma Release Response after a Second COVID-19 Booster Vaccination

**DOI:** 10.3390/vaccines10071163

**Published:** 2022-07-21

**Authors:** Katharina Grikscheit, Holger F. Rabenau, Zahra Ghodratian, Marek Widera, Alexander Wilhelm, Tuna Toptan Grabmair, Sebastian Hoehl, Emily Layer, Fabian Helfritz, Sandra Ciesek

**Affiliations:** 1 Institute for Medical Virology, University Hospital Frankfurt, Goethe University Frankfurt, 60596 Frankfurt, Germany; katharina.grikscheit@kgu.de (K.G.); rabenau@em.uni-frankfurt.de (H.F.R.); zahra.ghodratian@kgu.de (Z.G.); marek.widera@kgu.de (M.W.); a.wilhelm@em.uni-frankfurt.de (A.W.); tuna.toptangrabmair@kgu.de (T.T.G.); sebastian.hoehl@kgu.de (S.H.); 2 Health Protection Authority of the City of Frankfurt am Main, 60313 Frankfurt am Main, Germany; layer.emily@googlemail.com; 3 Bürgerhospital Frankfurt, Nibelungenallee 37-41, 60318 Frankfurt am Main, Germany; f.helfritz@buergerhospital-ffm.de; 4 German Centre for Infection Research (DZIF), Partner Site Frankfurt, 60596 Frankfurt, Germany; 5 Fraunhofer Institute for Molecular Biology and Applied Ecology, Branch Translational Medicine and Pharmcology, 60596 Frankfurt am Main, Germany

**Keywords:** SARS-CoV-2, variants of concern, neutralizing antibodies

## Abstract

The emergence of SARS-CoV-2 Omicron subvariants prompted countries to call for accelerated booster vaccinations to limit disease and transmission. Here, we characterized correlates of protection over time after the second booster or after Omicron BA.1 infection comparing variants of concern (VOCs). Sera from subjects before and two and seven weeks after the second booster or after Omicron infection were examined for the level of Spike receptor-binding-domain (RBD)-specific antibodies. Furthermore, neutralizing antibodies (nABs) were characterized in in vitro neutralization assays comparing the variants of concern Alpha, Beta, Delta, and Omicron BA.1 and BA.2 against the ancestral strain B.1. Here, the second booster resulted in an increase in anti-RBD-IgG-antibodies, remaining nearly constant over time, accompanied by an increase in nABs against B.1 and the VOCs Alpha, Beta, Delta, and Omicron BA.1 and BA.2. However, compared to B.1, the neutralizing capacity against the Omicron subvariants remained low and was limited after the second booster vaccination. This indicates that antibody-mediated protection against infection with this VOC is unlikely, as evidenced by the fact that three individuals of our study cohort became infected with Omicron BA.1 after the second booster. T cell activation was measured by interferon-gamma release assays in a subgroup of subjects and was increased in all subjects tested after the second booster vaccination, correlating with the amount of Spike-specific antibodies. In subjects with Omicron BA.1 breakthrough infection, a significant increase in nABs to all VOCs studied was observed independently of booster vaccinations. Taken together, our data indicate that a second booster or Omicron BA.1 infection mediate a substantial increase in anti-Spike IgG antibodies; however, infection with Omicron BA.1 induced a stronger increase in neutralizing antibodies against the different VOCs

## 1. Introduction

Since the end of 2020, different COVID-19 vaccines have been licensed, produced, and distributed to the population in Germany as the main measure of protection against severe disease and containment of the pandemic [1]. Vaccination and natural infection induce a humoral and cellular immune response [2]. Diagnostic assays measuring these effects serve as valuable tools to evaluate the protection efficacy. Particularly, induction of antibodies neutralizing the virus are critical for the protective humoral immune response and the levels of these nABs have been reported to correlate with the severity of COVID-19 [3,4]. These antibodies are substantially directed against the viral surface protein Spike and decreasing titers over time lead to reduced protection (“immune waning”) [5,6,7].

During the ongoing pandemic, various SARS-CoV-2 lineages and variants have emerged of which the WHO classified some as variants of concern (VOCs). While the first VOC Alpha (B.1.1.7) [8] has been defined mainly by a highly increased transmissibility, others such as Beta (B.1.351) [9,10], Delta (B.1.617) [11,12], and Gamma (P.1) [13] revealed dominant immune escape capacities. At the end of 2021, the variant Omicron (B.1.1.529) [14] was identified and has since spread rapidly around the globe. Omicron is characterized by a multitude of mutations, particularly in the Spike sequence, and had already diversified into several sublineages at the time of this study (BA.1, BA.2 [15], and BA.3 [16]). Studies support the notion that Omicron results in milder infections and less severe disease outcome [17]; however, health systems face consequences due to high patient numbers resulting from high transmissibility and immune escape, leading to infections in vaccinated and recovered patients. While biopharmaceutical companies are working on current strain-specific vaccines, all currently approved and administered vaccines are based on ancestral virus strains [2].

While in 2020/21 the recommendations for licensed primary vaccination series were continuously adjusted, two doses of mRNA-based vaccines represented the most common vaccination regime in Germany [1]. With increasing awareness of immune escape and waning, many countries rapidly announced booster vaccinations by the end of 2021 [17,18]. In this study, we use the term booster for any vaccine dose after the completed primary vaccination series according to the WHO [19].

In anticipation, some German health care providers started to administer employees with these first booster vaccinations at a time when many citizens had not yet completed their primary vaccination series. As this group consists mainly of health care workers with a high risk of exposure and transmission, second booster vaccinations were recently provided to this group during the Delta wave in winter 2021, immediately preceding the Omicron wave. Some countries, including Israel, administered second booster vaccinations on a larger scale to its inhabitants [20]. A report from an ongoing clinical trial in Israel was recently published and endorsed an official recommendation for elderly citizens, vulnerable individuals, and health care workers in January 2022, which the German institutions subsequently followed [20].

Here, we evaluated a cohort of non-immunosuppressed individuals consisting mainly of health care workers before and after second booster vaccination over time and analyzed the dynamics of serum antibody production and the activity of nABs against different VOCs. Furthermore, these data were evaluated in parallel with the findings of immunocompetent, healthy subjects who had recently recovered from a SARS-CoV-2 Omicron BA.1 infection.

## 2. Materials and Methods

### 2.1. Collection of Serum Samples

Participants were recruited either at the Bürgerhospital Frankfurt or University Hospital Frankfurt, Germany between January–March 2022. Peripheral blood was drawn in subjects prior, 2, or 7.5 weeks after the second booster and prepared for serological assays (Supplementary Materials Figure S1, Table S1).An additional group included 17 subjects after the 1st booster vaccination followed by an Omicron BA.1 infection (Supplementary Materials, Table S2). The inclusion criteria for all subjects were: >18 years, completed primary vaccination series and first booster with licensed vaccination scheme; no immunosuppression; and no SARS-CoV-2 infection prior to Omicron. For sera isolation, samples were centrifuged at 2000× *g*, room temperature for 10 min. Prior to the neutralization assay, samples were incubated at 56 °C for 30 min for complement inactivation.

### 2.2. Cell Culture

Reagents for cell culture were purchased from Sigma (St. Louis, MO, USA). Establishment of the A549-A+T cell line stably co-expressing ACE2 and TMPRSS2 is described in [21]. Cells were cultivated in Minimum Essential Medium supplemented with 10% FCS, 4 mM L-glutamine, 100 IU/mL of penicillin, and 100 µg/mL of streptomycin at 37 °C and 5% CO_2_.

### 2.3. Virus Propagation and SARS-CoV-2 Variants

SARS-CoV-2 isolates were propagated and collected as described in [22]. In brief, cell culture supernatant was collected after infected Caco-2 cells showed cytopathic effects, aliquoted, and stored at −80 °C. The median tissue culture infective dose (TCID_50_) was applied to determine the titers as described in [12]. All handling with SARS-CoV-2 was performed under biosafety level 3 conditions (Institute for Medical Virology, Frankfurt, Germany). The following SARS-CoV-2 virus variants were used in this study: B.1 (FFM7/2020), MT358643 [23]; Alpha (B.1.1.7) (FFM-UK7931/2021), MZ427280; Beta (B.1.351) (FFM-ZAF1/2021), MW822592 [9]; Delta (B.1.167.2) (FFM-IND8424/2021) MZ31514 [12]; Omicron BA.1 (B.1.1.529a (EPI_ISL_6959868); and Omicron BA.2 (B.1.1.529b) (EPI_ISL_6959871) [24].

### 2.4. Anti-Spike IgG Assay

The presence of SARS-CoV-2-specific anti-Spike (receptor-binding domain (RBD)) IgG antibodies was assessed using the Abbott SARS-CoV-2 IgG on the Abbott Alinity platform (Abbott GmbH, Wiesbaden, Germany) according to the manufacturer’s recommendations. Results are expressed as standardized binding antibody units (BAU/mL).

### 2.5. Neutralization Assay

Heat-inactivated sera were serially diluted (1:2) in MEM/1%FCS, 4 mM L-glutamine, 100 IU/mL of penicillin, and 100 µg/mL of streptomycin. All specimens were tested in duplicates. Sera dilutions were incubated with 4000 TCID_50_/mL of SARS-CoV-2 variants for 1 h at 37 °C. Antibody/virus mix was then added to A549-A+T cells and CPE formation was monitored after 72 h using a light microscope with complete CPE reduction as defined as neutralization. To control the reproducibility between the assays, we added a negative control (serum drawn prior to 2020) and a positive control (serum with a defined titer). For each experiment, we performed a backwards titration to verify the input virus and all virus strains were tested in parallel.

### 2.6. Interferon Gamma Release Assay (IGRA)

The interferon-gamma release assay (IGRA) was applied to quantitate IFN-gamma release by SARS-CoV-2-specific T cells and to assess hereby cellular immunity to SARS-CoV-2 (Euroimmun, Luebeck, Germany). In brief, in the first step, a specific T cell stimulation was performed with antigens based on the S1-domain of SARS-CoV-2 Spike. To this end, 500 μL of heparinized blood was added and incubated for 22–24 h at 37 °C. Then, interferon-gamma release was detected by an automated quantitative ELISA (Quan T-Cell ELISA, Euroimmun) using plates coated with anti-INF-gamma antibodies. The assay was carried out according to the manufacturer’s instructions and measured in mIU/mL.

### 2.7. Statistics

Graph Pad Prism (version 9.3.1, GraphPad Software) was used for statistical analysis. Statistical differences between groups were calculated using unpaired, non-parametric Mann–Whitney tests if not otherwise indicated. Adobe Illustrator (Adobe) was used for the figure design.

## 3. Results

### 3.1. Characterization of the Study Cohort after the Second Booster Vaccination

**Figure 1:**
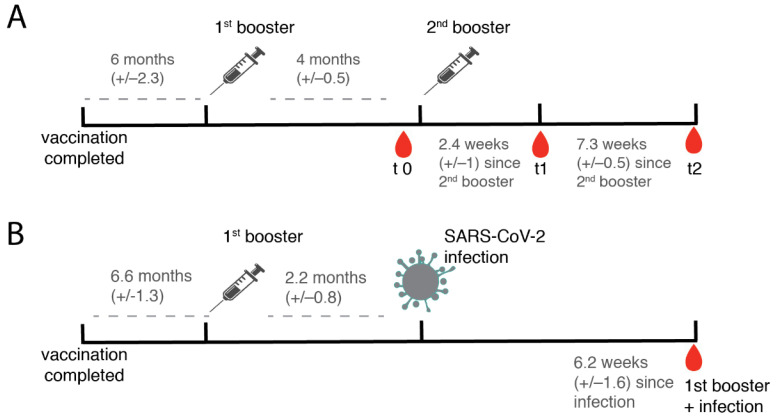
Study characteristics. (**A**) Overview of the vaccination scheme and sampling time points in a cohort after the second booster. Within the study group, 3 individuals contracted SARS-CoV-2 between t1 and t2 at unknown time points and are not indicated in the figure. (**B**) SARS-CoV-2 Omicron BA.1 infection after the first booster. The subjects were infected with SARS-CoV-2 on average 2.2 months after the first booster. Blood was drawn 6.2 +/−1.6 weeks after the infection and is thus considered time matched to t2 in A. For an overview of the vaccination scheme of the subjects, please refer to Table S1.

We investigated a cohort of 26 healthy subjects with a mean age of 49.6 years who received a second booster vaccination against SARS-CoV-2 4+/−0.5 months after the first booster (Figure 1A). Vaccination regimes were not uniform among the cohort and included individuals who received mRNA and vector vaccines (Supplementary Materials Table S1). As the Johnson and Johnson vaccine has been licensed as a single-dose regimen, we defined the first booster as any subsequent vaccination after the licensed primary vaccination and the second booster as any additional dose of vaccine (as defined by the WHO, [19]).

Blood was collected before (t0, N = 14), at 2.4+/−1 weeks (t1, N = 26), and at 7.3+/−1.4 weeks (2, N = 15) after the second booster dose and antibody responses were analyzed (Figure 1A, Supplementary Materials Figure S1, Table S1). During the study period, three individuals contracted an infection with the Omicron variant BA.1).

In addition, convalescent sera were examined from 17 subjects who had had a documented infection with Omicron BA.1 at a period comparable to t2 in the other subjects (7.3+/−1.4 weeks versus 6.2+/−1.6 weeks) (Figure 1B, Supplementary Materials Table S2). All enrolled SARS-CoV-2-recovered subjects had mild symptomatic infections.

### 3.2. Anti-Spike IgG Antibody Levels after the Second Booster Vaccination

**Figure 2:**
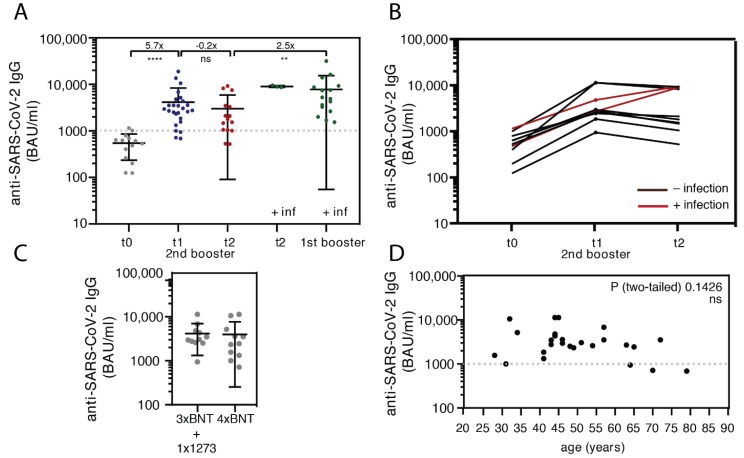
Characterization of the anti-Spike IgG antibody response after the second booster vaccination or after SARS-CoV-2 infection after booster vaccinations. (**A**) Anti-SARS-CoV-2 IgG levels were determined and indicated as binding antibody units per milliliter (BAU/mL) in samples before the second booster (t0), 2 weeks (t1), 7.3 weeks (t2), or infection after the first or second booster (unpaired, non-parametric, Mann–Whitney, t0 vs. t1 *p* < 0.0001 (indicated as ****); t1 vs. t2 ns, t2 vs. 1st booster + infection *p* < 0.0063 (indicated as **); error bars mean+/−SD), ns = non-significant. (**B**) Individual responses of 8 individuals after the second booster vaccination and 2 after the second booster followed by a SARS-CoV-2 infection are plotted. (**C**) Anti-SARS-CoV-2 IgG levels dissected between the most common immunization schemes in Germany in 2021 (3× BNT162b2 and 1X mRNA-1273 and 4x BNT162b2) (unpaired, non-parametric, Mann–Whitney, ns—not significant, error bars mean+/−SD). (**D**) The correlation between age and IgG levels does not show any significance (linear regression—not significant). Abbreviations: BNT = BNT162b2; 1273 = mRNA-1273.

Anti-SARS-CoV-2 Spike-RGB IgG antibodies determined by means of chemiluminescence microparticle-immunoassays were analyzed and plotted, comparing the different time points (Figure 2A, Supplementary Materials Table S1). As shown in Figure 2A, the second booster resulted in a significant increase in SARS-CoV-2-specific anti-RBD-antibodies two weeks after vaccination compared to t0 (Figure 1A, 5.7-fold (range 3–27, *p* < 0.0005)). Antibody levels neither differed between the vaccination regimes (Figure 2C) nor with the age of the subjects (Figure 2D). A slight decrease of 20.5% in the IgG levels was detected 7.3+/−1.4 weeks after the second booster. The progression of antibody levels was similar in the eight individually recorded subjects (Figure 2B). However, the IgG levels in individuals who experienced a SARS-CoV-2 infection after the second booster vaccination (red lines) continued to increase during the course (Figure 2B). Thus, natural SARS-CoV-2 infection after the second booster vaccination induces high IgG levels (Figure 2A,B). The primary routes of entry of SARS-CoV-2 are the oral and nasal cavities and, therefore, mucosal immunity is critical to protect against infection [25]. In order to estimate whether the IgG levels in saliva correspond to the level of IgGs in serum, we collected the saliva of 10 specimens of subjects from our cohort at t2 after the second booster vaccination. Supplementary Material Figure S2 demonstrates a correlation between the serum and salivary IgG levels and two SARS-CoV-2 recovered subjects (red dots) appear within the highest range.

Finally, we examined the levels of SARS-CoV-2-specific antibodies in 17 time-matched subjects who had SARS-CoV-2 infection 2.2+/−0.8 months after the first booster and who had blood drawn 6.2+/−1.6 weeks after diagnosis, a period similar to t2 (7.3 +/−1.4 weeks, Figure 2B, Supplementary Materials Table S2). Figure 2A shows that Omicron BA.1 infection after the first booster led to a larger increase in anti-RBD IgGs compared to a second booster vaccination (2.5 fold, *p* < 0.0323).

### 3.3. Booster Vaccination Elicited Neutralizing Antibodies against SARS-CoV-2 Variants

**Figure 3:**
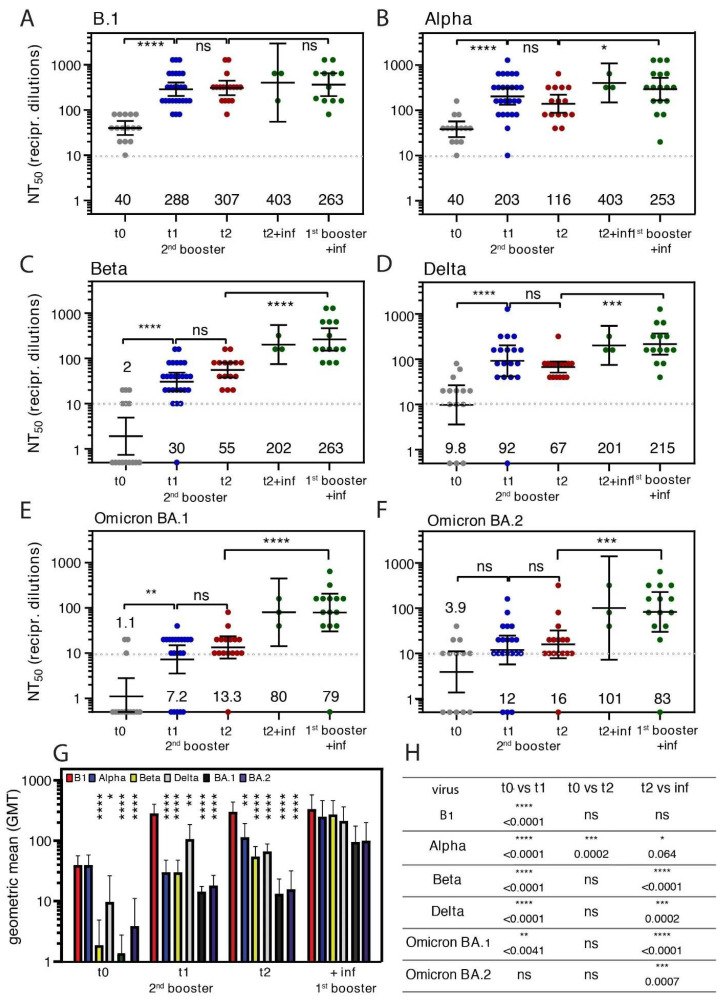
Characterization of neutralization titers after the second booster or infection against different VOCs. Neutralization assays were conducted using the authentic SARS-CoV-2 strains B.1 (**A**), Alpha (**B**), Beta (**C**), Delta (**D**), and Omicron BA.1 (**E**) and Omicron BA.2 (**F**) using sera from recipients after the second booster vaccinations at t0 (N = 15), t1 (N = 26) and t2 (N = 15). The analysis also included sera from individuals after breakthrough infections after the second booster (N = 3) and first booster (N = 17) (unpaired, non-parametric, Mann–Whitney, error bars = geometric mean with 95% CI). Statistics are summarized in (H). (**G**) Graph depicting the geometric means of tested groups as indicated by the color code (unpaired, non-parametric, Mann–Whitney). (**H**) Summary of the statistical analysis of A–F (unpaired, non-parametric, Mann–Whitney). Asterisks indicate significance: **** *p* < 0.0001; *** *p* < 0.001; ** *p* < 0.01; * *p* > 0.01, ns = non-significance.

High levels of nABs are considered an indicator of protection against SARS-CoV-2. However, antibodies directed against SARS-CoV-2 are produced in varying quality and quantity after vaccination or recovery and these levels decrease over time [5]. Here, we evaluated the activity of vaccine-elicited sera after the second booster vaccination to neutralize in vitro SARS-CoV-2 infection using the parental virus isolate B.1 harboring the D614G mutation against the VOCs Alpha, Beta, Delta, and Omicron BA.1 and BA.2. nABs against B.1 were detected in all but one individual 4 months after the first booster vaccination (t0) (Figure 3A). After second 2nd booster (t1), nABs against B.1 increased 7-fold (t1) and remained at a comparable level after 7.2 weeks (t2) (Figure 3A). Elicited antibodies neutralized the Alpha variant as described previously [10] but did not further increase after the second booster (Figure 3B). Three months after the first booster (t0), only 20–60% of sera showed neutralization activity against the immune-escape-associated VOCs Beta, Delta, and Omicron subvariants BA.1 and BA.2, which increased after the second booster vaccination (Figure 3C–F). However, in a direct comparison of VOCs, the neutralization efficiency against Beta, BA.1, and BA.2 was poorest three months after the first booster but higher after the second booster and was then comparable to the neutralization against Alpha (Figure 3G). However, the neutralization of vaccine-elicited antibodies against all VOCs remained significantly lower compared to B.1 (Figure 3G). Overall, we observed an increase in nABs after the second booster, but only low titers of antibodies were detected, especially for Beta, BA.1, and BA.2, resulting in insufficient protection against infection with these variants in vitro (Figure 3C,E,F).

### 3.4. Neutralizing Antibodies against Diverse Variants of SARS-CoV-2 after Omicron BA.1 Infection

During the Omicron wave, breakthrough infections were frequently reported. Thus, we further evaluated individuals who contracted an infection either after the first or the second booster and assessed their level of nABs against different SARS-CoV-2 variants. Infection with BA.1 resulted in significant enhancement of nABs 6.2 weeks after infection against B1, Alpha, Beta, Delta, BA.1, and BA.2 (Figure 3B–F). Overall, sera from subjects after booster vaccinations and consecutive Omicron infection exhibited the most efficient neutralization activity against a broad range of variants, which was significantly increased compared to a second booster alone (Figure 3G,H). Only neutralization of the parental variant B.1 was not further increased (Figure 3A).

### 3.5. Evaluation of Interferon-Gamma Release after the Second Booster Vaccination

**Figure 4:**
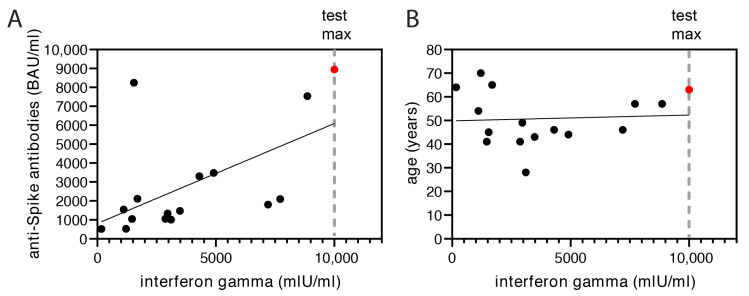
Interferon-gamma release of T cells after the second booster vaccination. Interferon-gamma release assays were performed with 16 subjects 7.3 weeks after the second booster vaccination (Table S1). Interferon gamma concentration is measured in mlU/mL (mIU—milli international units). Results were plotted against (**A**) the anti-Spike IgGs (BAU/mL) (linear regression—R = 0.3) or (**B**) age (years) (liner regression—not significant).

Memory T cell responses have been described to be essential for protection against severe outcomes of SARS-CoV-2 infection [26]. We used an interferon-gamma release assay to indirectly evaluate antigen-specific T cell activation and assessed 16 subjects 7.3 weeks after the second booster vaccination. T cell activation was detected against SARS-CoV-2 in all tested subjects which weakly correlated with the levels of anti-Spike antibodies in serum (Figure 4A) but not with age (Figure 4B). The subject with a recent infection after the second booster vaccination (red dot) had the highest score in the test.

## 4. Discussion

Here, we examined the immune responses of vaccinated subjects at 2 and 7 weeks after a second booster dose. Limitations of our study include the small number of subjects, with ten subjects covering all three time points (Supplementary Materials Table S1). Using this cohort, our data show that the second booster led to (i) an intermediate increase in anti-Spike-RBD antibodies peaking 2 weeks after vaccination that remained at a similar level after 7 weeks, and (ii) resulted in an activated T cell response in the subjects in our study. Furthermore, compared to the parental strain B1, the second booster vaccination resulted in a weaker increase in nABs against the VOCs Beta, Delta, and Omicron BA.1 and BA.2.

The waning of IgG levels after COVID-19 immunization is universal, which prompted recommendations for additional booster vaccinations in many countries [27,28]. In line with an ongoing clinical trial evaluating the vaccine safety and efficacy of mRNA-vaccines in health care workers in Israel [20], we found that the IgG levels after the second booster vaccination increased ~6–10-fold 2 weeks after administration. However, this elevation is considered moderate due to high baseline levels after 4–5 months after the first booster compared to IgG levels measured directly after immunization (~>30-fold depending on the study) [29].While independent studies often have limited comparability, it becomes clear that long-term investigations regarding immune waning after different immunization schemes in healthy subjects are important to judge long-term vaccination schemes after the pandemic phase.

A further challenge of the pandemic is the emergence of novel immune escape variants of SARS-CoV-2 such as the Omicron lineage [6,14]. We observed that sera from subjects who contracted SARS-CoV-2 Omicron BA.1 after the first but also after the second booster exhibited significantly elevated IgG levels against SARS-CoV-2 Spike-RBD compared to individuals that had been vaccinated with a second booster. Increased levels of anti-Spike-RBD-IgG after breakthrough infections preceded by basic immunization or booster vaccinations have been described earlier [30,31], thereby indicating different and/or additive mechanisms of the immune responses after infection compared to vaccination [32]. However, detailed information about this phenomenon is lacking.

Samples from subjects after the second booster vaccination and reconvalsecent sera after the first and second booster were further examined for the activity of nABs against VOCs, including the Omicron variants BA.1 and BA.2. We demonstrated that level of nABs against the Omicron subvariants were severely reduced compared to the parental B.1 variant at all time points despite high levels of anti-Spike IgGs after the second booster. This is in agreement with a recently published study that compared neutralizing antibodies after the second booster in a large clinical cohort comparing wildtype SARS-CoV-2 against the VOCs Delta and Omicron [20]. While they found a 13× lower activity against Omicron compared to the wildtype strain 2 weeks after the booster, here, we detected a 40× lower neutralizing activity against Omicron BA.1 compared to B.1. However, we consider this comparable despite the different study protocols and the different sizes of the cohorts [33].

Reduced neutralization of the Omicron subvariants was also described for reconvalescent sera of individuals infected with other SARS-CoV-2 variants [24,32]. Here, we did not detect a significant difference between the Omicron sublineages BA.1 and BA.2 in sera after the second booster or subjects who recovered from Omicron BA.1 as observed in reports assessing sera after the first booster vaccination [28].

Furthermore, nABs against all variants, including Delta and Alpha, were detected in individuals after infection with SARS-CoV-2 Omicron BA.1 after the first booster vaccination. Intriguingly, Omicron BA.1 infection strongly enhanced nABs against the Beta variant compared to the second booster vaccination alone (Figure 3C). Both variants and Beta share a mutation on position N501Y in the RBD of the Spike protein that has been assigned to strong immune escape characteristics [34]. These results suggest protection against infection with related variants such as BA.2 and possibly Beta after exposure to Omicron BA.1 infection in vaccinated or reconvalescent individuals [24,34]. Our data support the notion that a second booster restores the levels of anti-Spike antibodies and, therefore, counteracts immune waning. However, the protection against in vitro infections, as measured with neutralization assays, is limited against immune-escape-associated VOCs such as Omicron BA.1 and BA.2 [6,32]. In consequence, the finding that booster vaccinations led to increased but low levels of nABs suggests that at-risk individuals such as the elderly who show prominent immune waning could benefit from regular vaccine doses [35]. The positive effect of regular vaccination of the healthy population might be restricted, as currently, the infection rate of Omicron remains high and the burden of disease lower than in previous variants.

Furthermore, studies that thoroughly compare cellular responses such as the induction of T cell immunity after the first and second booster vaccination are of high relevance to identify whether booster vaccinations remain beneficial to maintaining cell-mediated immunity [36].

## Data Availability

Not applicable.

## Supplementary Materials

The following supporting information can be downloaded at: https://www.mdpi.com/article/10.3390/vaccines10071163/s1, Figure S1: Graphical summary of groups used in the study. N = female vs male; age and IgG = mean. For more details refer to Table S1 and S2; Figure S2: Correlation between anti-SARS-CoV-2 Spike IgGs measured in serum using SARS-CoV-2 IgG II Quant assay (Abott) and Anti-SARS-CoV-2 ELISA in saliva (Euroimmun). N = 10, red dots = infected individuals after second booster vaccination (Table S1), Correlation—two-tailed, non-parametric. Asteriks (**) in-dicate *p* < 0.01; Table S1: Characteristics of the study population after a second booster vaccination; Table S2: Characteristics of the study population after a booster vaccination followed by Omicron BA.1 infection.
