# Supplementary material for: Characterization of the Antibody and Interferon-Gamma Release Response after a Second COVID-19 Booster Vaccination
Source: Vaccines (Basel). 2022 Jul 21;10(7):1163. doi: 10.3390/vaccines10071163 (PMC9323888; doi:10.3390/vaccines10071163)
Supplement: Supplementary file 1 [file vaccines-10-01163-s001.zip › vaccines-1731714-supplementary.pdf]

Supplement:

Characterization of antibody and T-cell response after second COVID-19 booster vaccination

Supplement Methods:

Anti-Spike-IgG assay

Serum anti-Spike receptor-binding-domain (RBD) IgG antibody concentration was determined using the SARS-CoV-2 IgG-II Quant assay and the Alinity I device (Abott Diagnostics, Germany) with an analytical measurement range from 2.98–5680 binding antibody units per mL (BAU/mL).

Supplement Figures:

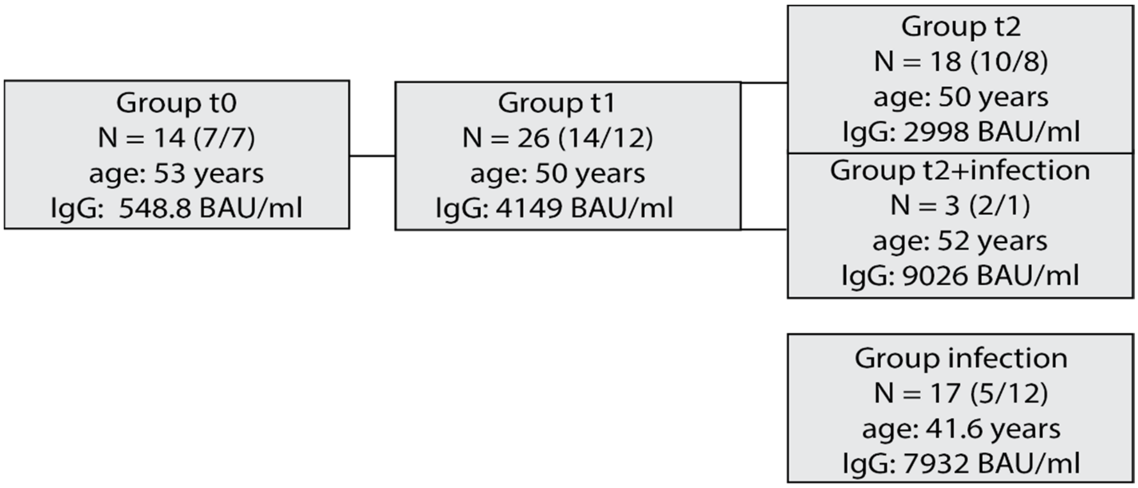

Supplement Figure S1: Graphical summary of groups used in the study. N=female vs male;age and IgG = mean. For more details refer to Table S1 and S2.

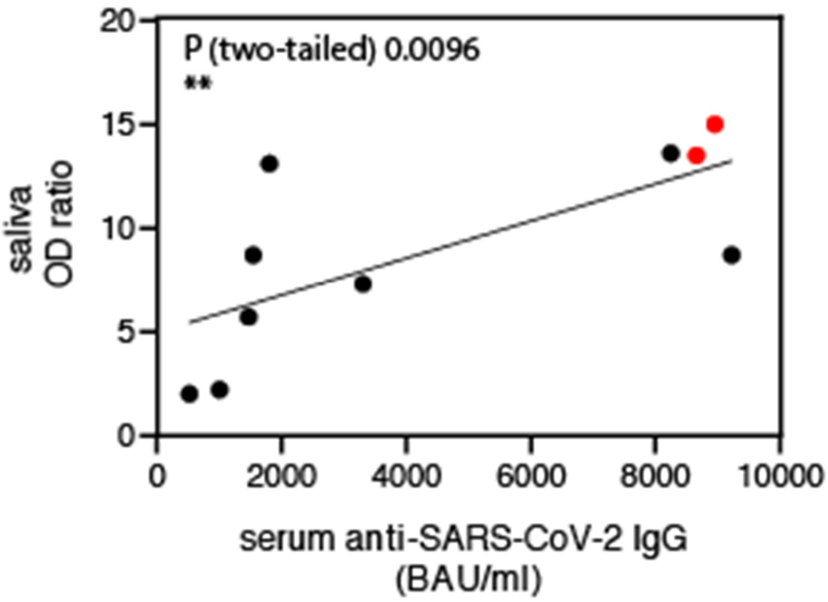

Supplement Figure S2: Correlation between anti-SARS-CoV-2 Spike IgGs measured in serum using SARS-CoV-2 IgG II Quant assay (Abott) and Anti-SARS-CoV-2 ELISA in saliva (Euroimmun). N=10, red dots = infected individuals after 2nd booster vaccination (Table S1), Correlation – two-tailed, non-parametric. Asteriks (**) indicate p<0.01

Supplement Table S1: Characteristics of the study population after a second booster vaccination

No	age	sex	regime	1st booster (b1)	2nd booster (b2)	Diff. b1/b2 (months)	t1 weeks since b2	t2 weeks since b2	t3 weeks infection/b2	IgG t1 (BAU/ml)	IgG t2 (BAU/ml)	IgG t3 (BAU/ml)	IGRA (mlU/ml)
1	45	m	BNT/BNT	BNT	1273	4	2	8.6		1018.2	13640	8242.2	1542
2	43	f	BNT/BNT	BNT	1273	4	2	8.6		638.8	2761.4	1468	3485
3	79	m	ChAd/ChAd	BNT	1273	3.6	2			125.4	691.4		
4	72	f	ChAd/ChAd	BNT	1273	3.6	2			262.1	3541.8		
5	44	m	BNT/BNT	BNT	1273	3	2	8.6	6	1155.7	*4799.1*	9477.8	
6	63	f	ChAd/ChAd	BNT	1273	3.6	2	8.6	4	*485.2*	*2708.8*	8948.4	>10.000
7	41	m	J&J	BNT	1273	3.6	2	8.6		199.7	1851.4	1044.7	1463
8	64	m	BNT/BNT	BNT	1273	5.4	2	8.7		124.7	942.3	521.7	169
9	46	f	BNT/BNT	BNT	1273	4.3	2	6.0		630.3	2955.4	1802.4	7206
10	44	f	BNT/BNT	BNT	1273	4.8	2.6	6.6		407.1	18910	9219.7	
11	54	m	BNT/BNT	BNT	BNT	5	2	6.0		535	2597.9	1542.7	1113
12	65	m	BNT/BNT	BNT	1273	3.6	5	8.9		796.2	2437.9	2114.2	1690
13	43	f	BNT/BNT	BNT	BNT	4.3	2			611.5	3473.8		
14	48	f	BNT/BNT	BNT	1273	3.6	1.6			692.8	2523.4		
15	51	m	BNT/BNT	BNT	1273	3.6	4				3049.2		
16	44	f	BNT/BNT	BNT	1273	3.6	2	6.0			4333.4	3478.1	4911
17	57	f	BNT/BNT	BNT	1273	4.3	2	6.0			6814.1	7536.5	8863
18	34	m	BNT/BNT	BNT	1273	4.3	2.7.				5188.1		
19	28	f	BNT/BNT	BNT	BNT	4.1	1.6	5.6			1560.8	1009.2	3113
20	46	f	BNT/BNT	BNT	BNT	4.3	5	8.9			3579.5	3302	
21	41	m	BNT/BNT	BNT	BNT	3.6	1.9	5.9			1321.7	1057.5	4300
22	31	f	BNT/BNT	BNT	BNT	4.3	2.6				1004.3		2873

23	70	f	BNT/BNT	BNT	BNT	4.1	5	8.9			718.7	532.7	1213
24	57	m	BNT/BNT	BNT	BNT	3.6	2	6			3527.4	2100	7717
25	32	f	BNT/BNT	BNT	BNT	4.3	2.3				10620.7		
26	49	f	BNT/BNT	BNT	BNT	4.4	1.6	5.6	1.1		2330.8	8652.8	2960
Mean<br>**(range)**	49.6<br>(28-79)					4<br>(3.0-5.4)	2.5<br>(1.6-5)	7.3<br>(5.6-8.9)	3.7<br>(1.1-6.0)	548.8<br>(124-1156)	4149<br>(691.4-18910)	4003<br>(512-9478)	3914<br>(169-10.000)

Supplement Table S2: Characteristics of the study population after a booster vaccination followed by Omicron BA.1 infection

No	age	sex	regime	1st booster (b1)	vacc/b1 (months)	b1/infection (weeks)	analysis after infection (weeks)	IgG t2 (BAU/ml)
27	59	m	1273/1273	1273	5.6	0.7	4.7	14000
28	31	f	BNT/BNT	BNT	8.0	9.1	7.9	10.537.8
29	31	m	BNT/BNT	BNT	7.6	9.4	7.6	4292.6
30	45	m	BNT/BNT	BNT	8.4	1.4	6.1	5257
31	54	m	BNT/BNT	BNT	7.0	1.4	6.7	31840
32	28	f	BNT/BNT	BNT	6.2	0.4	6.6	*7461.8*
33	63	m	BNT/BNT	BNT	5.9	1.7	8.4	4923.4
34	27	m	1273/1273	BNT	9.2	3.0	5.9	1544.9
35	32	m	1273/1273	BNT	5.0	1.7	6.1	7535.7
36	22	m	BNT/BNT	BNT	5.2	4.7	5.4	2637.4
37	50	f	BNT/BNT	BNT	5.5	5.0	4	3612.1
38	50	m	1273/1273	BNT	8.4	6.9	4	16240.0
39	52	m	BNT/BNT	BNT	2.0	3.0	3.9	3285.6
40	51	m	BNT/BNT	BNT	5.5	6.3	4.4	1998.5
41	30	m	BNT/BNT	BNT	5.4	2.9	6.7	9324.3
42	31	f	BNT/BNT	1273	7.3	-4.9	7.3	8667
43	51	f	BNT/BNT	BNT	5.6	1.4	9.2	1687.21
Mean **(range)**	41.6 (22-63)				6.3 (2-7.2)	3.2 (-4.9-9.4)	6.2 (3.9-9.2)	7932 (1545-31840)

Abbreviations: BNT = BNT162b2, Pfizer-BioNtech vaccine; 1273 = mRNA-1273, Moderna vaccine, JJ = Johnson&Johnson vaccine, ChAD = ChAdOx-1S = AstraZeneca vaccine
